# Box breathing and prolonged exhalation reduces markers of physiological stress reactivity in response to a virtual trier social stress test

**DOI:** 10.1016/j.cpnec.2026.100360

**Published:** 2026-06-25

**Authors:** Matthew J. McAllister, M. Hunter Martaindale, Nate Sutton, Judith P. Andersen

**Affiliations:** a Metabolic & Applied Physiology Laboratory, Department of Health & Human Performance, Texas State University, San Marcos, TX, 78666, USA; b ALERRT Center, Texas State University, San Marcos, TX, 78666, USA; c Department of Psychological and Brain Sciences, University of Toronto Mississauga, Toronto, Mississauga, ON, Canada

**Keywords:** Alpha amylase, Cortisol, Secretory immunoglobulin A, Slow breathing, Heart rate variability

## Abstract

Acute exposure to psychological stress activates the sympathetic “fight or flight” response, increasing heart rate, respiratory rate, and metabolic activity. High-stress occupations such as law enforcement, firefighting, and military service regularly involve exposure to intense stress and may benefit from interventions that mitigate physiological and psychological stress responses. Slow-breathing techniques such as box breathing and prolonged exhalation have been shown to reduce perceived stress and certain physiological markers; however, their relative effectiveness under acute stress conditions remains unclear.

In this randomized controlled trial, sixty-six participants (n = 66) were randomly assigned to normal breathing (NB; n = 22), prolonged exhalation (PE; n = 22), or box breathing (BOX; n = 22) prior to completing a modified, virtual Trier Social Stress Test (TSST). Markers of subjective and physiological stress were collected before and after the TSST, including state anxiety (SAI), heart rate (HR), heart rate variability (HRV; RMSSD, SDNN), and salivary concentrations of alpha-amylase (sAA), secretory immunoglobulin A (SIgA), and cortisol, as well as cognitive performance (Stroop color-word task and mental arithmetic). Utilizing a linear mixed model with a random subject intercept, the TSST elicited significant increases in HR, SAI, sAA, and SIgA. Both PE and BOX breathing attenuated post-stressor increases in HR, SAI, and sAA compared to NB, indicating that brief breathing practice can buffer acute physiological stress responses. HRV, Cortisol, and cognitive performance were statistically unchanged across the three conditions. These findings support the short-term effectiveness of both box breathing and prolonged exhalation for reducing subjective and peripheral physiological markers of stress.

## Highlights

• This was the first study to examine the impact of box breathing and prolonged exhalation on physiological markers of stress reactivity using a virtual trier social stress test.
• The virtual trier social stress test significantly increased physiological markers of stress.
• Both box breathing and prolonged exhalation significantly attenuated physiological stress reactivity.

## 1 Introduction

Acute psychological stress typically triggers coordinated changes in brain and body systems that are generally adaptive, enabling individuals to meet situational demands such as heightened physical and cognitive performance [1]. However, when stress responses escalate into sustained *fight or flight* state, characterized by increased heart rate, respiratory rate, blood flow, and substrate utilization, they may impair rather than enhance cognition and performance [2]. Repeated or chronic activation of these systems contributes to oxidative stress, inflammation, dyslipidemia, and hypertension, increasing the risk of cardiometabolic disease [1,[3], [4], [5]].

Exposure to both acute and chronic stressors is particularly relevant for high-stress occupations, such as firefighters, military personnel, and law enforcement officers, who face elevated risks for stress-related cardiometabolic and psychological disorders [6,7]. In these professions, maintaining optimal physical and cognitive function depends on the ability to effectively modulate stress responses in real time [8,9]. Consequently, identifying low-cost, easily implemented strategies to mitigate stress is of significant occupational and public health interest.

A range of interventions have been examined for their potential to regulate stress, including stress inoculation or exposure training, which can reduce stress reactivity with only a few exposure sessions [10,11]. Nutritional supplements such as caffeine or L-tyrosine have also demonstrated modest effects on cognitive performance under stress [12,13]. However, in terms of mitigating the physiological impact of stress, these nutrients have demonstrated marginal effects. In contrast, slow-breathing interventions have emerged as a practical and accessible approach with growing empirical support for their physiological and psychological benefits [[14], [15], [16]].

Slow or paced breathing, typically defined as fewer than 10 breaths per minute, can increase parasympathetic activation, reduce physiological arousal, and support emotional regulation in high-stress situations [[15], [16], [17]]. Promising research with first responders suggests that slow-breathing interventions can enhance performance and health, reduce physiological stress, and support better decision-making [[17], [18], [19], [20]]. Slow breathing offers advantages over many other interventions: it requires no equipment, can be performed virtually anywhere, and can be integrated into both acute stress and recovery contexts.

Slow breathing has been employed in various forms as a psychological and physiological stress mitigation strategy [16,21]. A range of slow breathing interventions have been applied in high-stress occupational contexts, as summarized by Andersen et al. [22]. Of relevance to the present study are techniques designed for short-duration application. These include “box” breathing, characterized by an equal-ratio of 4:4:4:4 cycle (4-s inhale, 4-s pause, 4-s exhale, 4-s pause), and the “reset breath,” which emphasizes a deep inhalation and a prolonged exhalation through pursed lips [17]. The prolonged exhalation technique used in the present study is characterized by periodic physiological sighs (i.e., single deep breaths with extended exhalation interspersed with normal breathing) rather than a sustained reduction in overall respiratory rate [20]. Variations of box breathing, also popularized as ‘tactical or combat breathing’ among first responder populations [23] have demonstrated reduced salivary biomarkers of stress from 5 min of 4:2:4 (4-s inhale, 2-s pause, 4-s exhale) as well as 4:2 (4-s inhale, 2-s exhale) [14]. Both techniques (i.e., box breathing and the reset breath) have demonstrated reductions in stress biomarkers (e.g., salivary alpha-amylase) and improvements in mood and composure following stressful events [14,18]. However, relatively few studies have directly compared different breathing methods under controlled laboratory stress paradigms.

The physiological effects of breathing interventions are best understood when examined under controlled stress conditions. Laboratory-based paradigms allow researchers to observe both the psychological and physiological components of the stress response and evaluate how interventions modulate these processes. Among these paradigms, the Trier Social Stress Test (TSST) is one of the most widely used and validated methods for eliciting both subjective anxiety and physiological stress markers [[24], [25], [26]]. Heart rate variability (HRV), which reflects the variation in time between successive heartbeats, is a noninvasive index of autonomic regulation that captures the dynamic interplay between sympathetic and parasympathetic activity. While slow breathing has been shown to influence cardiovascular and autonomic markers such as heart rate and HRV, findings have varied depending on respiratory rate, duration, and timing relative to the stressor [15,27]. Despite this variability, existing evidence generally supports slow breathing to impact autonomic activity during stressful events, reflected by improved recovery following exposure to stressors.

However, key gaps in the existing literature remain. Most existing studies have examined either subjective stress, HRV, or salivary biomarkers in isolation. Few have integrated multiple indices to examine both psychological and physiological stress responses concurrently. Moreover, little research has compared ultra-short breathing interventions (<6 min total) that could realistically be deployed in operational contexts where extended relaxation training is impractical. Therefore, the purpose of the present randomized controlled trial was to examine the effects of two methods of slow breathing (i.e., standard box breathing and prolonged exhalation) on markers of physiological and subjective stress during a virtual TSST. We hypothesized that both breathing interventions would attenuate increases in salivary stress biomarkers (sAA, SIgA, cortisol), heart rate, and state anxiety relative to the normal breathing control condition. Given the brevity of the interventions utilized, effects on HRV and cognitive performance were considered exploratory and were not formally hypothesized.

## 2 Methods

### 2.1 Subjects and design

This study was a randomized controlled trial (clinical trial #NCT06986135) where subjects (n = 66) were randomly assigned to perform normal breathing [(NB); n = 22: 8 males, 14 females], prolonged exhalation [(PE) n = 22: 8 males, 14 females] or box breathing [(BOX) n = 22: 9 males, 13 females] prior to participating in a virtual/modified TSST (see section 2.2.1 below). Data collection occurred after clinical trials registration. Randomization was conducted via a random generator (random.org), where each subject was assigned a number one-three (1 = NB; 2 = BOX; 3 = PE). Following the initial six randomizations, the generation was then replicated for the remaining subjects to maintain a balanced assignment. Subjects were recruited from {{blinded for peer review}} University campus. Inclusion criteria: ages 18-39 years. Exclusion criteria were assessed via health history questionnaire and a member of the research team and included: a known history of cardiovascular or metabolic disease, diagnosed psychological conditions (i.e., anxiety, depression, ADHD), or medications for these conditions. Subjects were also required to be free from any major stressors in the last 30 days such as the birth of a child, abortion, or divorce. Subjects reported use of oral contraceptives (Yes = 8; No = 58) and nicotine products (Yes = 2; No = 64). Subjects were also asked immediately prior to starting the protocol whether they had fasted for at least 4 h (Yes = 62; No = 4). Given the small number of participants reporting nicotine use (n = 2) and non-fasted status (n = 4), these variables were not expected to meaningfully influence the results; however, they are acknowledged as minor limitations.

All procedures were reviewed and approved by the Texas State University institutional review board (IRB # 9665), and participants provided written informed consent prior to participation. Testing sessions occurred between 10:00 and 16:00 h to minimize diurnal variation in stress biomarkers. It should be noted that time of day has been shown to impact heart rate, salivary α-amylase (sAA), secretory immunoglobulin A (SIgA), and cortisol concentrations (in different ways) [28,29]. Because of this, a 40 min resting period was implemented prior to starting the TSST in an attempt to standardize stress markers to baseline levels.

### 2.2 Experimental procedures

All testing occurred within the Metabolic & Applied Physiology Laboratory at Texas State University. Each subject was asked to arrive at least 4 h fasted. Prior to participation, subjects provided written informed consent and completed a health history questionnaire prior to arrival to ensure they qualified. Upon arrival to the lab, subject body mass and height were recorded using a digital scale (Rice Lake Weighing System, Rice Lake, WI, USA) and a stadiometer (213; SECA, CA, USA). Subjects then rinsed their mouths out with filtered water, performed a brief 60 s familiarization trial to learn the cognitive challenge, and rested for 10 min. Following the initial 10-min rest period, baseline measurements were collected. Subjects performed their assigned breathing method twice: 1) for 5 min immediately prior to the TSST, and 2) for 1 min during the TSST (between the presentation and cognitive challenge). At all other times, subjects were told to breathe normally. The cognitive challenge was conducted 35 min prior to the TSST, as well as post presentation portion of the TSST (immediately after completing 1 min of their assigned breathing).

**Fig. 1:**
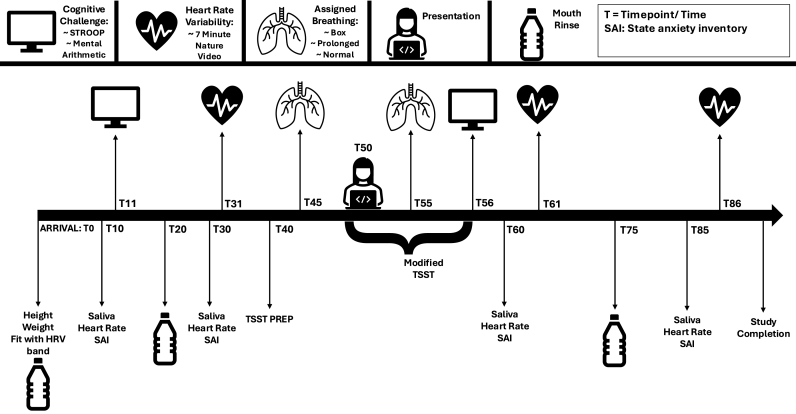
Overview of experimental procedures. "T" = time in minutes after arrival.

A saliva sample, heart rate (HR), and state anxiety inventory (SAI) were obtained at four timepoints: 1) 40 min prior to starting the TSST; 2) 10 min prior to TSST; 3) immediately post TSST; 4) 25 min post TSST. Heart rate variability (HRV) was measured at three timepoints: 1) 10 min prior to TSST, 2) post TSST, and 3) 25 min post TSST. An overview of all procedures is shown in Fig. 1.

#### 2.2.1 Trier social stress test

This study incorporated a modified, virtual TSST as a psychological stressor. Ten minutes prior to the TSST, subjects were given notepaper and a pencil/pen and instructed to prepare a 5-min presentation convincing two judges that they were strong candidates for a job of interest to them. They were allowed 5 min to take notes and prepare for a speech. A virtual TSST was selected for standardization purposes since the research team did not have personnel to consistently act as judges during each session for each participant.

Subjects presented virtually via Zoom in front of a prerecorded video of two judges for another 5 min. The video was shown to the participants instead of judges being present for the testing. The Zoom call video included a male and female interviewer wearing professional attire and maintaining neutral expressions while taking notes. Subjects were informed that the judges would not respond to questions or any attempts at interaction. Immediately upon completion of the presentation, subjects performed 1 min of their assigned breathing task before completing the cognitive challenge described below.

The present protocol represented a modified version of the standard TSST in several respects [24]. First, participants presented to a pre-recorded rather than a live judge panel, reducing personnel requirements and increasing standardization across sessions. Second, the preparation period was shortened to 5 min relative to the 10-min period used in the original protocol. Third, two interruptions were introduced during the stressor for the assigned breathing intervention — once immediately prior to the presentation and once between the presentation and cognitive challenge. These modifications were necessary given the study design but represent meaningful deviations from the validated protocol that are acknowledged in the limitations section.

#### 2.2.2 Cognitive challenge

The cognitive challenge, previously used by our team [30,31], consisted of a rapid Stroop color-word test and a mental arithmetic task, both of which were administered via a computer using e-Prime 2 software (Psychology Software Tools, Inc., Pittsburgh, PA, USA). A 60 s familiarization trial was provided prior to the first cognitive challenge (at baseline). Immediately following the familiarization, subjects then participated in a 2-min Stroop challenge, followed by 2 min of the mental arithmetic. For the Stroop task, subjects were shown a .5 s display of a word on the computer (i.e., blue, green, yellow, or red). The word displayed on the screen was written in a conflicting color (i.e., the word red shown in blue font). Subjects were instructed to identify the color of the word displayed as quickly and accurately as possible using color-coded keyboard keys. The mental arithmetic calculations consisted of simple addition and subtraction problems involving single, double-, and triple-digit numbers (e.g., 120 minus 13). Subjects were given 10 s to respond using the numeric keypad; a buzzer sounded following incorrect answers.

#### 2.2.3 Breathing intervention

The assigned breathing intervention was performed for 5 min immediately before the presentation for the TSST, and again for 1 min during the TSST (immediately after the presentation and before the cognitive challenge). For the Normal Breathing (NB) group, subjects were instructed to breathe as they normally would. The box breathing (BOX) and prolonged exhalation (PE) groups were described the breathing method in advance and followed a visual prompt timer that instructed them to “inhale”, “hold”, and “exhale” when appropriate. Instructions and a visual demonstration (<1 min) for the breathing technique were provided immediately before starting the breathing task. The visual count timer was created using Visual Studio Code (Microsoft Windows, Redmond, WA, USA). The BOX group followed a 4:4:4:4 breathing ratio; thus, the subject was instructed to perform a 4 s inhale, 4 s hold, 4 s exhale, 4 s hold, repeat. Additionally, the participants assigned to the PE group performed a prolonged exhalation breath consisting of a deep inhalation through the nose for 3 s, followed by a slow, controlled exhalation through pursed lips for 6 s. This single breath was performed once every 30 s, with participants breathing normally in the intervals between prompted breaths, resulting in 2 PE breaths every minute. This approach is consistent with prior research examining the effects of sighing and periodic prolonged exhalation on stress reactivity [20]. In terms of instruction, participants were told they would be assigned to one of the three breathing groups. The breathing intervention groups were explained in the informed consent. The only instruction given to participants included which breathing group they were assigned to, and they were told they would follow a countdown timer to follow and to match their inhalation and exhalation timing with the visual timer. A researcher provided a demonstration of practicing the breathing technique using the timer. A researcher was in the room for the entirety of the intervention to observe the respiratory ratio to ensure the participant was keeping up with the tempo. Moreover, researchers monitored protocol adherence in real time using a Go Direct® Respiration Belt (Beaverton, OR, USA) to verify breathing rate and rhythm. Participants did not report any difficulties or discomfort while adhering to the breathing interventions.

#### 2.2.4 Heart rate variability (HRV) and heart rate (HR)

Beat to beat HR and HRV measures were collected utilizing Polar H10 chest strap monitors (Polar Electro Ltd, Kempele, Finland). The Polar H10 detects R peaks with an internal sampling frequency of approximately 1000 Hz, yielding RR-intervals with 1 ms resolution. During HRV measures, subjects watched a 7-min standardized nature video and data were recorded throughout the entire duration. Upon arrival at the laboratory, subjects were fitted with the polar chest straps and data were collected via an iPad using the HRV Trace app (Ibernetics, LLC; Eugene, OR, USA). HRV data were collected for a 7 min duration at each of the three following timepoints: 1) 10 min prior to TSST, 2) post TSST, and 3) 25 min post TSST. Raw data from the HRV Trace app were exported to the Kubios software app (Kubios Oy; Kuopio, Finland) where HRV data were visually inspected for outliers. Automatic noise detection was set at ‘medium’ and automatic beat correction before interpretation. The following variables were analyzed: Root mean square of successive differences (RMSSD), and standard deviation of NN intervals (SDNN). HR data were collected four times using the Polar Teams App (Polar Electro Ltd, Kempele, Finland): 1) 40 min prior to starting the TSST; 2) 10 min prior to TSST; 3) immediately post TSST; 4) 25 min post TSST.

#### 2.2.5 State anxiety inventory

State anxiety was measured using the State Anxiety Inventory (SAI) short form (6-items; [32,33]). Subjects rated how calm, tense, or worried they felt on a 4-point scale depending on how strongly they felt for each feeling. Subjects subsequently marked these feelings as “very likely” or “not at all” to calculate the cumulative score. SAI data were collected four times: 1) 40 min prior to starting the TSST; 2) 10 min prior to TSST; 3) immediately post TSST; 4) 25 min post TSST.

#### 2.2.6 Saliva sampling and analysis

Subjects provided a saliva sample (∼1.5 mL) 4 times throughout the study which were concurrent with HR measures: 1) 40 min prior to starting the TSST; 2) 10 min prior to TSST; 3) immediately post TSST; 4) 25 min post TSST. Saliva samples were collected using a passive drool method where subjects tilted their head forward and passively guided saliva into a 2 mL cryovial via a saliva collection aid (Salimetrics, PA). Subjects rinsed their mouths with water ∼10 min prior to providing each sample. Samples were immediately placed in a −80°C freezer. Saliva samples were collected concurrent with each HR and SAI four total times: 1) 40 min prior to starting the TSST; 2) 10 min prior to TSST; 3) immediately post TSST; 4) 25 min post TSST. Samples were shipped overnight via FedEx on dry ice to a laboratory for analysis (Salimetrics SalivaLab, CA). Samples were thawed and centrifuged prior to being assayed for duplicate concentrations of sAA, SIgA, and cortisol. The intra-assay and inter-assay % coefficient is reported as follows: 5.4 and 4.7% for sAA, 4.6 and 6.0% for cortisol, and 5.6 and 8.7% for SIgA.

### 2.3 Statistical analysis

All statistical procedures were conducted using R version 4.6.0. To examine potential changes across time for HR, SAI, HRV metrics, saliva stress markers (sAA, SIgA, cortisol), and cognitive performance, linear mixed models (LMMs) were used. Each LMM included treatment, timepoint, and their interaction as fixed effects, a random intercept for subject, and a first-order autoregressive (AR(1)) within-subject correlation structure when three or more timepoints were available. For outcomes with only two timepoints (i.e., cognitive performance), the AR(1) parameter is not identifiable and the model reduced to a random-intercept LMM. Denominator degrees of freedom for the F-tests were obtained using the containment method in nlme. A secondary constrained longitudinal data analysis (cLDA), which assumes a shared baseline mean across randomized groups, was fit as a sensitivity check for each outcome. Type III F-tests are reported for the fixed effects, and per-timepoint marginal means with standard errors and Tukey-adjusted pairwise contrasts were obtained via the emmeans package. For outlier detection, data demonstrating standardized residuals ±3.0 were identified and excluded from analysis. Effect sizes are presented as partial eta squared (ηp^2^). As a sensitivity analysis, the primary mixed model for each time-of-day-sensitive marker (sAA, cortisol, SIgA, and heart rate) and for SAI was re-fit adding the mean-centered TSST start clock time as a covariate, entered both as a main effect and as an interaction with the timepoint, to assess whether within-window variation in session start time contributed to the observed effects.

As exploratory analyses, the association between the change in sAA and the change in SAI was examined using a within-person coupling model across all four timepoints. sAA was separated into a between-person component (each participant's mean across the protocol) and a within-person, person-mean-centered component, and both were entered as predictors of SAI with a random subject intercept.

## 3 Results

**Fig. 2:**
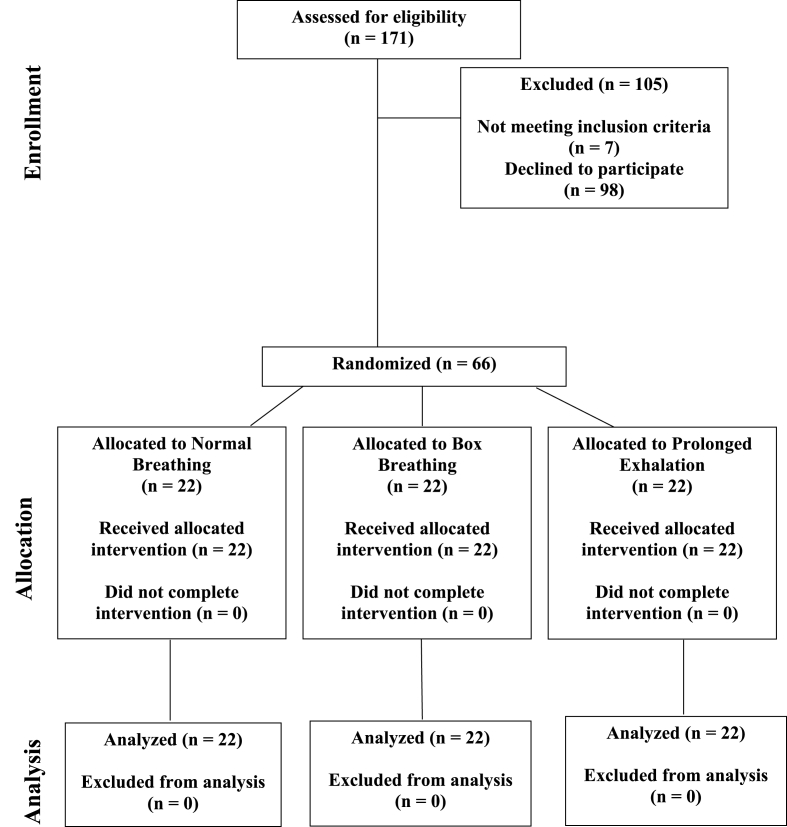
Consort diagram.

Sixty-six subjects (n = 66: 25 males; 41 females; Table 1) completed experimental testing. An overview of enrollment, screening, and allocation is shown in Fig. 2. With respect to linear mixed model results, all F scores and *p* values are shown in Table 2.

**Table 1:** Subject descriptive characteristics.

Treatment	Male/Female	Height (cm)	Weight (kg)	Age (years)
**NB**	8 Male	171.2 ± 8.0	69.9 ± 10.4	19.9 ± 1.3
	14 Female			
**PE**	8 Male	171.6 ± 7.6	69.0 ± 15.6	20.5 ± 2.9
	14 Female			
**BOX**	9 Male	173.1 ± 10.6	74.1 ± 18.7	21.5 ± 2.1
	13 Female			

NB = normal breathing group; PE = prolonged exhalation group; BOX = box breathing group.

**Table 2:** Linear mixed model results for all outcomes.

Variable	Effect	df	F	p	ηp^2^
Salivary alpha-amylase (sAA)	Treatment	2, 61	4.46	.016	.127
	Time	3, 179	6.91	<.001	.104
	Treatment x Time	6, 179	2.80	.013	.086
Salivary cortisol	Treatment	2, 61	.67	.514	.022
	Time	3, 181	9.37	<.001	.134
	Treatment x Time	6, 181	.53	.786	.017
Salivary IgA (SIgA)	Treatment	2, 63	2.11	.129	.063
	Time	3, 182	18.08	<.001	.230
	Treatment x Time	6, 182	1.53	.170	.048
Heart rate (HR)	Treatment	2, 63	.81	.449	.025
	Time	3, 189	10.09	<.001	.138
	Treatment x Time	6, 189	8.27	<.001	.208
State anxiety inventory (SAI)	Treatment	2, 63	.96	.389	.030
	Time	3, 189	10.95	<.001	.148
	Treatment x Time	6, 189	2.29	.037	.068
HRV: RMSSD	Treatment	2, 57	.76	.475	.026
	Time	2, 109	1.33	.270	.024
	Treatment x Time	4, 109	.78	.538	.028
HRV: SDNN	Treatment	2, 58	.59	.560	.020
	Time	2, 110	1.80	.170	.032
	Treatment x Time	4, 110	.96	.432	.034
Mental arithmetic: correct	Treatment	2, 58	.20	.818	.007
	Time	1, 56	.51	.479	.009
	Treatment x Time	2, 56	.52	.598	.018
Mental arithmetic: missed	Treatment	2, 57	.05	.954	.002
	Time	1, 55	.69	.411	.012
	Treatment x Time	2, 55	.18	.836	.006
Mental arithmetic: response time	Treatment	2, 58	1.19	.311	.040
	Time	1, 58	6.92	.011	.107
	Treatment x Time	2, 58	.19	.831	.006
Stroop: correct	Treatment	2, 57	.41	.665	.014
	Time	1, 53	.16	.687	.003
	Treatment x Time	2, 53	.60	.555	.022
Stroop: missed	Treatment	2, 54	1.80	.176	.062
	Time	1, 50	.02	.888	.000
	Treatment x Time	2, 50	.93	.403	.036
Stroop: response time	Treatment	2, 59	.66	.519	.022
	Time	1, 59	4.53	.038	.071
	Treatment x Time	2, 59	1.00	.373	.033

Note. F-tests are Type III tests (computed with sum-to-zero contrasts so main effects are averaged over the levels of the other factor) from a linear mixed model with treatment, timepoint, and their interaction as fixed effects, a random intercept for subject, and a first-order autoregressive within-subject correlation structure (random intercept only for 2-timepoint cognitive outcomes). ηp^2^ (partial eta-squared) = (F × dfnum)/(F × dfnum + dfden). LMM = linear mixed model; AR(1) = first-order autoregressive; df = degrees of freedom; HR = heart rate; SAI = state anxiety inventory; HRV = heart rate variability; RMSSD = root mean square of successive differences; SDNN = standard deviation of normal-to-normal (NN) intervals; sAA = salivary alpha-amylase; SIgA = secretory immunoglobulin A.

### 3.1 Salivary stress markers

**Fig. 3:**
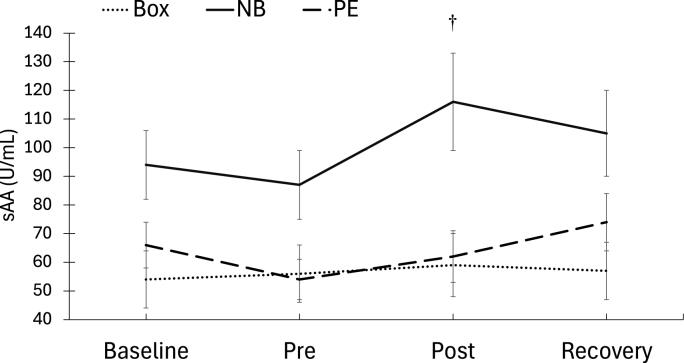
Changes in salivary alpha amylase (sAA) concentrations across time and between groups. † indicates a significant increase in the NB compared to both PE and box breathing conditions. NB = normal breathing, Box = box breathing and PE = prolonged exhalation group. Data are shown as mean ± standard error.

**Fig. 4:**
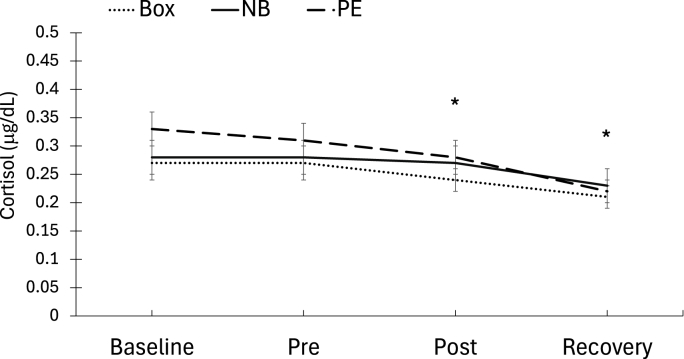
Changes in salivary cortisol concentrations across time and between groups. ∗ indicates a significant reduction from the previous timepoint. NB = normal breathing, Box = box breathing and PE = prolonged exhalation group. Data are shown as mean ± standard error.

**Fig. 5:**
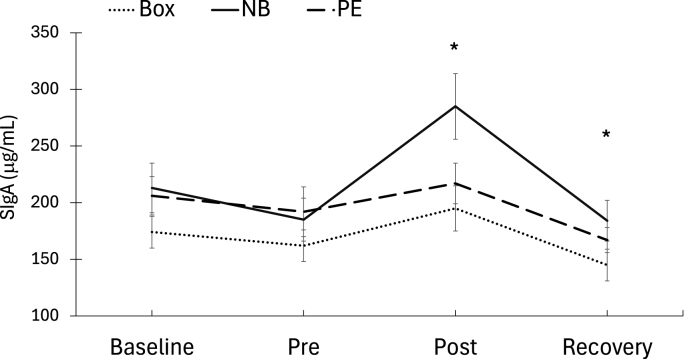
Changes in salivary secretory immunoglobulin A (SIgA) concentrations across time and between groups. ∗ indicates a significant change from the previous timepoint. NB = normal breathing, Box = box breathing and PE = prolonged exhalation group. Data are shown as mean ± standard error.

Regarding saliva stress markers, a significant treatment × timepoint interaction was found for sAA (F_(6, 179)_ = 2.80, p = 0.013, ηp^2^ = .086). Pairwise contrasts indicated that the NB group showed a significant pre-to-post increase in sAA (+27.7 U/mL, p < 0.001), whereas neither the BOX (+1.9, p = 0.80) nor PE (−1.2, p = 0.87) group showed a change. At post-TSST, sAA was significantly higher in NB than in both BOX (+60.0 U/mL, p = 0.002) and PE (+56.0 U/mL, p = 0.004; Fig. 3). For cortisol, a significant main effect of timepoint was observed (F_(3, 181)_ = 9.37, p < 0.001, ηp^2^ = .134) reflecting a gradual decline across the session consistent with diurnal variation rather than a stress response. Neither treatment (F_(2, 61)_ = .67, p = 0.51) nor the treatment × timepoint interaction (F_(6, 181)_ = .53, p = 0.79) was significant (Fig. 4). In relation to SIgA, a significant main effect of timepoint was found (F_(3, 182)_ = 18.08, p < 0.001, ηp^2^ = .230), but neither the treatment main effect (F_(2, 63)_ = 2.11, p = 0.13) nor the interaction (F_(6, 182)_ = 1.53, p = 0.17) reached significance. The significant timepoint effect reflects an overall increase in SIgA from baseline to post-TSST across the full sample. Because neither the treatment main effect nor the treatment × timepoint interaction was significant, the SIgA response to the TSST did not differ across breathing conditions (Fig. 5).

### 3.2 Heart rate and state anxiety inventory

**Fig. 6:**
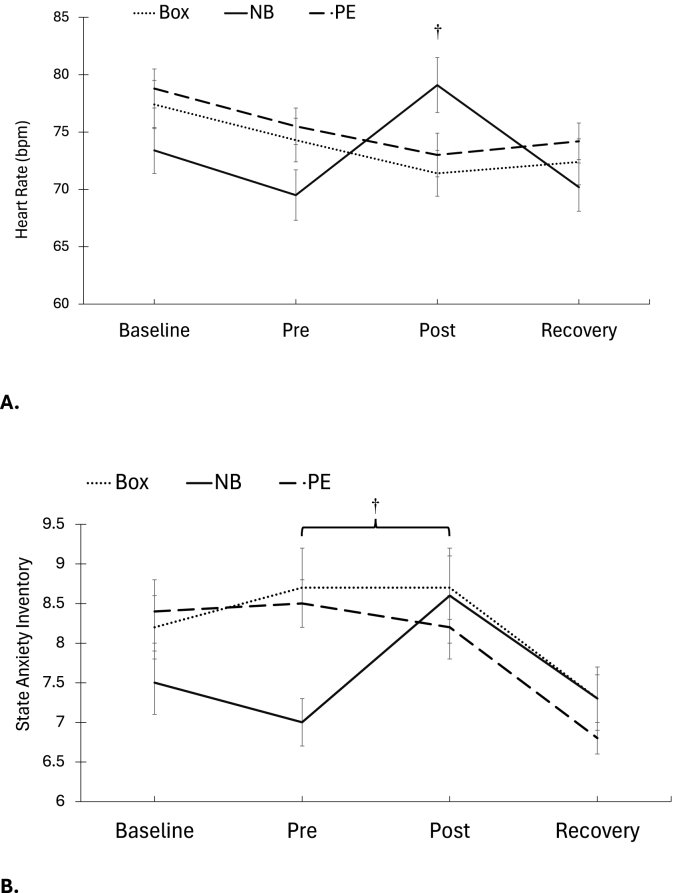
Changes in heart rate (HR, A) and state anxiety inventory (SAI; B) across time and between groups. † indicates a significant interaction and differences between groups at selected timepoints. From the pre-TSST timepoint (immediately before the TSST) to immediately post-TSST (i.e., time 2 to time 3), only the NB group showed a significant increase in SAI (+1.6, p < 0.001); whereas neither BOX (+.05, p = 0.91) nor PE (−.2, p = 0.59) changed. At the pre-TSST timepoint, NB was lower than both BOX (p = 0.019) and PE (p = 0.049), before the breathing intervention was performed. NB = normal breathing, Box = box breathing and PE = prolonged exhalation group. Data are shown as mean ± standard error.

Regarding HR, a significant treatment × time interaction was found (F_(6, 189)_ = 8.27, p < 0.001, ηp^2^ = .208). The three groups did not significantly differ at the pre-TSST baseline (−40 min; all pairwise p > 0.24). The NB group demonstrated a significant pre-to-post HR increase (+4.4 bpm, p = 0.009), whereas both BOX (−6.4 bpm, p < 0.001) and PE (−5.3 bpm, p = 0.002) showed significant pre-to-post decreases. At post-TSST, NB HR was significantly higher than BOX (+7.8 bpm, p = 0.047); the NB vs PE post-TSST contrast was in the same direction but did not reach significance (+4.5 bpm, p = 0.35). Mean HR data are shown in Fig. 6A.

Regarding SAI data, a significant treatment × timepoint interaction was noted (F_(6, 189)_ = 2.29, p = 0.037, ηp^2^ = .068). Pairwise contrasts indicated that the three groups did not significantly differ at the −40 min baseline (all Tukey-adjusted p > 0.29). From the pre-TSST timepoint (immediately before the TSST) to immediately post-TSST (i.e., time 2 to time 3), only the NB group showed a significant increase in SAI (+1.6, p < 0.001); whereas neither BOX (+.05, p = 0.91) nor PE (−.2, p = 0.59) changed. Between-group contrasts revealed no significant differences immediately post-TSST (NB vs BOX, p = 0.99; NB vs PE, p = 0.78) or at the +25 min recovery; the only significant between-group difference occurred at the pre-TSST timepoint, where NB was lower than both BOX (p = 0.019) and PE (p = 0.049), before the breathing intervention was performed. The significant interaction therefore reflects divergent SAI trajectories across the groups (a post-stressor increase in NB that was absent in the slow breathing groups), rather than NB exhibiting higher absolute anxiety than the breathing groups at peak stress. Mean SAI data are shown in Fig. 6B.

### 3.3 Heart rate variability metrics

No significant interactions or main effects were observed for either RMSSD or SDNN (RMSSD: treatment F_(2, 57)_ = .76, p = 0.47; time F_(2, 109)_ = 1.33, p = 0.27; interaction F_(4, 109)_ = .78, p = 0.54; SDNN: treatment F_(2, 58)_ = .59, p = 0.56; time F_(2, 110)_ = 1.80, p = 0.17; interaction F_(4, 110)_ = .96, p = 0.43), indicating no change in HRV across the testing session in any group.).^1^ Mean HRV data are shown in Table 3.

**Table 3:** Heart rate variability data.

Treatment	Timepoint	RMSSD	SDNN
Normal	Pre	64.7 ± 35.0	67.3 ± 26.1
	Post	76.8 ± 40.3	72.6 ± 28.5
	Recovery	63.1 ± 28.0	64.4 ± 22.4
Prolonged	Pre	68.2 ± 33.5	68.0 ± 19.8
	Post	75.4 ± 31.4	76.6 ± 23.8
	Recovery	75.7 ± 34.7	72.5 ± 22.4
Box	Pre	62.0 ± 21.6	62.2 ± 18.3
	Post	59.2 ± 27.2	61.0 ± 18.4
	Recovery	64.0 ± 28.9	69.0 ± 26.9

Data are shown as mean ± SD. RMSSD = Root Mean Square of Successive Differences; SDNN = Standard Deviation of NN Intervals.

### 3.4 Cognitive performance metrics

No significant interactions or treatment main effects were observed for any of the cognitive performance variables: mental arithmetic correct, mental arithmetic missed, mental arithmetic response time, Stroop correct, Stroop missed, or Stroop response time (all p > 0.10). A small time main effect was observed for Stroop response time (F_(1, 59)_ = 4.53, p = 0.038, ηp^2^ = .071), with post-TSST response times slightly lower than pre-TSST values across all groups, consistent with practice or familiarization. A significant time main effect was also observed for mental arithmetic response time (F_(1, 58)_ = 6.92, p = 0.011, ηp^2^ = .107), reflecting slower responses post-TSST; given the absence of any treatment effect or interaction, this most likely reflects task-general factors rather than the breathing intervention. Collectively, these results indicate no impact from the breathing interventions on cognitive performance, and minimal impact possibly from the TSST. Cognitive performance data are shown in Table 4 (mental arithmetic) and Table 5 (Stroop).

**Table 4:** Mental arithmetic performance data.

Treatment	Timepoint	MA Correct	MA Missed	MA Response Time
Normal	Pre	9.5 ± 2.4	2.0 ± 1.7	4501.7 ± 1286.0
	Post	9.2 ± 2.2	1.9 ± 1.2	4699.7 ± 1150.0
Prolonged	Pre	9.1 ± 2.0	2.2 ± 1.5	4725.8 ± 1011.2
	Post	9.5 ± 1.9	2.0 ± 1.7	5053.2 ± 1107.0
Box	Pre	9.4 ± 2.0	2.2 ± 1.7	4237.2 ± 1022.9
	Post	9.8 ± 1.5	1.9 ± 1.4	4592.8 ± 960.8

Data are shown as mean ± SD. MA = Mental Arithmetic. MA Correct and MA Missed are count data. MA Response Time presented in milliseconds.

**Table 5:** Stroop performance data.

Treatment	Timepoint	Stroop Correct	Stroop Missed	Stroop RT
Normal	Pre	29.9 ± 1.9	1.1 ± 1.0	622.3 ± 66.0
	Post	30.2 ± 1.9	.9 ± 1.3	597.5 ± 77.5
Prolonged	Pre	30.6 ± 1.8	.5 ± .6	643.1 ± 88.4
	Post	29.7 ± 2.6	.9 ± .9	625.5 ± 75.7
Box	Pre	29.7 ± 2.5	1.4 ± 1.8	616.6 ± 73.1
	Post	29.5 ± 2.7	1.3 ± 1.7	614.9 ± 70.0

Data are shown as mean ± SD. RT = Response Time.

### 3.5 Sensitivity checks

Adjusting for TSST start time left the treatment × timepoint interaction essentially unchanged for every outcome (sAA: F_(6,179)_ = 2.80, p = 0.013 both within and without adjustment; HR: F_(6, 189)_ = 8.27, p < 0.001; cortisol was non-significant in both; SAI: F_(6, 189)_ = 2.29, p = 0.037), and the pattern of significant-per-timepoint group contrasts was preserved. The reported effects are therefore not attributable to within-window variation in session start time.

As an exploratory analysis, the association between the change in sAA and the change in SAI was examined to assess whether the physiological and subjective stress responses tracked one another. The two changes were not significantly associated. A within-person mixed model using all four timepoints confirmed the absence of coupling (standardized within-person slope = .03, 95% CI [−.07, .13]), with no difference across breathing groups (F_(2, 185)_ = 2.10, p = 0.13). Thus, although sAA and SAI each showed the expected treatment × timepoint pattern at the group level, individual changes in the two measures were largely independent, consistent with the well-documented dissociation between physiological and subjective indices of acute stress.

## 4 Discussion

The primary findings of this study demonstrate that both BOX and PE attenuated post-stressor increases in sAA, SIgA, SAI, and HR relative to the NB group. The NB group exhibited the expected physiological and psychological stress response, characterized by significant pre-to-post increases in HR, SAI, and sAA following the TSST; SIgA also increased over time across the full sample, although this increase did not differ by group. The slow breathing groups showed either no change or, for HR, a modest decrease in these markers. The genuine intervention effects (i.e., the treatment × timepoint interactions for sAA, HR, SAI, and the within-group divergence for SIgA) were robust. Cortisol did not show a stress response in any group, consistent with the modified virtual TSST protocol limitations discussed below.

Pairwise contrasts indicated that the three groups did not significantly differ in SAI at the −40 min baseline, but only the NB group showed a significant pre-to-post anxiety increase. The interaction reflects divergent trajectories. This pattern is consistent with both BOX and PE preventing the acute anxiety increase associated with the TSST, even if absolute SAI levels are similar across groups at any given moment.

Regarding heart rate, the NB group demonstrated the expected pattern of stress induced sympathetic activation (or reduction of parasympathetic activity) which was demonstrated by a significant increase in HR post-TSST. The slow breathing groups did not show a significant post-stressor HR increase. From a physiological standpoint, a transient increase in HR during a speech task is an adaptive response that supports performance demands, and the ability to recover rapidly may be more meaningful than peak HR per se. Whether the attenuation of HR response in the slow breathing groups reflects a beneficial buffering effect or a blunting of an adaptive response warrants further investigation in future studies examining performance outcomes alongside physiological stress markers.

The present findings align with prior research from Dillard et al. [14] who reported reduced sAA and no impact on SIgA concentrations from slow breathing in response to acute stress. Collectively, the comparable effects across different box breathing protocols (including the traditional 4:4:4:4 ratio used here and the modified protocols examined by Dillard et al.) suggest that the precise inhale-to-exhale ratio may be less critical than simply reducing overall respiratory rate and incorporating controlled, deep breathing. Finally, it is worth mentioning that while the breathing interventions in the present study, as well as Dillard et al., were conducted for a 5-min duration, the individuals in the PE group only conducted this once every 30 s, which may provide more real-world practicality or convenience. That is, from an applied perspective, the PE approach may offer practical advantages in operational environments due to its simplicity and minimal cognitive load, whereas box breathing may be more suitable for training or recovery contexts emphasizing deliberate focus and relaxation.

Neither cortisol nor SIgA showed significant treatment × time interactions. Cortisol showed a significant main effect of time, reflecting a gradual decline across the session consistent with normal diurnal variation rather than a stress-related increase, but no treatment effect or interaction. The absence of a cortisol response warrants discussion, as the TSST reliably elicits HPA axis activation in its standard form [24]. We attribute the lack of cortisol elevation to the shortened and interrupted nature of the present protocol relative to the original TSST. The standard TSST employs a 10-min uninterrupted stressor, whereas the present study used a 5-min preparation period with two interruptions for the breathing intervention. The interruptions, while necessary to the study design, likely attenuated the continuity of the stressor below the threshold required for a meaningful cortisol response. For SIgA, the significant main effects for time without an interaction suggest that SIgA fluctuations across the session were not differentially modulated by the breathing interventions, consistent with findings from Dillard et al. [14].

The absence of significant treatment × time interactions for HRV indices is best understood as a reflection of the measurement design rather than evidence that the breathing interventions had no effect on autonomic regulation. HRV was assessed during resting conditions at three timepoints (prior to, immediately following, and 25 min after the TSST) and was not measured during the breathing task itself. As such, any acute changes in cardiac vagal tone induced by the breathing interventions would not be expected to persist through the subsequent stressor and recovery periods in a manner detectable during resting measurement. Additionally, the relatively high baseline RMSSD values observed across all groups (range: 59-77) are consistent with a young, healthy sample, which may further limit the sensitivity of resting HRV measurement to detect stress- or intervention-related change in this population [34]. This represents a design limitation and precludes conclusions regarding the acute autonomic effects of either intervention. Future studies should measure HRV continuously throughout the breathing task and the subsequent stress protocol to capture the dynamic autonomic response to the intervention.

Cognitive performance was included as an exploratory outcome. No significant treatment × time interactions or treatment main effects were found for any cognitive performance variable. The only effect observed was a small time main effect for Stroop response time, with slightly faster post-TSST RTs across all groups, consistent with practice or familiarization rather than an intervention effect. Given the brevity of the breathing interventions and the lack of a formal hypothesis regarding cognitive outcomes, these results should be interpreted cautiously. Future studies with longer intervention durations and more sensitive cognitive assessments may be better positioned to detect meaningful effects.

The present study employed a modified virtual TSST in which participants presented to a pre-recorded judge panel via a simulated video conference. While the standard TSST typically employs live role players, virtual and semi-virtual adaptations have been previously examined with varying success (see Allen et al. [26]). Virtual reality-based assessments offer a practical method to utilize the TSST since it drastically reduces the personnel needed. However, similar speech tasks conducted in virtual reality environments have been suggested to be less effective compared to in person TSST assessments since a past study showed no significant increase in stress biomarkers despite significant increases in heart rate and blood pressure [35]. Importantly, the present paradigm successfully elicited significant increases in HR, SAI, sAA, and SIgA in the NB group, representing a coherent multi-system stress response across both peripheral physiological and subjective markers that supports the construct validity of this virtual adaptation. The absence of a cortisol response is more plausibly attributed to the shortened and interrupted nature of the protocol (specifically the 5-min preparation period and two breaks introduced by the breathing intervention) than to the virtual format per se, as cortisol elevation via HPA axis activation requires sustained, uninterrupted social-evaluative stress of sufficient duration [36]. To our knowledge, the present study is among the first to examine whether a pre-recorded judge panel is sufficient to elicit a stress response in a virtual TSST context, and the multi-marker stress response observed in the NB group suggests this approach has merit as a practical and scalable adaptation. Whether a live versus pre-recorded judge panel produces meaningfully different stress responses remains an open empirical question that future research should address directly, ideally with a within-subjects or matched design that includes cortisol as a primary outcome with an uninterrupted stressor protocol.

The present study has limitations that should be acknowledged. First, since subjects presented in front of a pre-recorded panel of judges, the researchers had to inform the subjects that the judges were instructed to not respond or interact in any way. Had subjects realized the judges were prerecorded, they may have perceived the evaluation as less socially threatening than traditional live-administration protocols. Data were not collected from the subjects related to whether they perceived the judges to be pre recorded. However, it should be noted that the version of the TSST introduced in this study successfully increased several markers of stress (SAI, sAA, SIgA, and HR), validating its use as a virtual adaptation. Additionally, the cognitive assessments used have not been fully validated for reliability and sensitivity to stress-induced change. Furthermore, measures of HRV were not taken during the ultra-brief breathing intervention and thus examining the immediate impact of the breathing on cardiorespiratory functionality was not possible. The present study also lacked an a priori power analysis to formally justify the sample size. With 25 males and 41 females distributed across three groups, the sample was insufficiently powered to examine sex as a potential moderator of breathing intervention effects on stress reactivity. Given well-documented sex differences in HPA axis reactivity and autonomic regulation [25], future studies should be explicitly powered to test sex as a moderating variable. Lastly, participants were not formally queried during debriefing regarding their awareness of the pre-recorded nature of the judge panel, precluding a formal manipulation check of the perceived social-evaluative threat. Future studies employing this virtual adaptation should incorporate such a check to confirm ecological validity.

## 5 Conclusions

In conclusion, this study supports the use of brief, slow-breathing techniques as effective, low-cost methods for reducing subjective and peripheral physiological responses to acute stress. Both box breathing and prolonged exhalation reduced heart rate, anxiety, and salivary stress biomarkers following exposure to a psychological stressor. However, neither technique produced significant changes in HRV, suggesting that short-term breathing practice may attenuate acute sympathetic activation and perceived stress without measurable shifts in autonomic modulation. Future research should investigate whether repeated or longer-term practice of these breathing methods leads to more robust improvements in autonomic flexibility and stress resilience. Moreover, studies comparing the operational utility of box breathing versus prolonged exhalation in acute, high-stress environments are needed to determine which technique is more effective and sustainable under real-world conditions. The present study did not have any measures of cognitive load to draw comparisons between the breathing interventions; however, both slow breathing interventions were effective at reducing multiple physiological markers of acute stress.

## Data accessibility statement

Data will be made available upon reasonable request. However, consent was not obtained by participants to make deidentified data publicly available.

## Funding

The authors disclose receipt of partial financial support for the execution of this project: U.S. DOJ – 10.13039/100013135Office of Community Oriented Policing Services (10.13039/100013135COPS Office) Award # 15JCOPS-24-GK-02506-PASS.

## CRediT authorship contribution statement

**Matthew J. McAllister:** Conceptualization, Data curation, Formal analysis, Funding acquisition, Investigation, Methodology, Supervision, Writing – original draft, Writing – review & editing. **M. Hunter Martaindale:** Conceptualization, Funding acquisition, Investigation, Methodology, Software, Writing – original draft, Writing – review & editing. **Nate Sutton:** Investigation, Methodology, Project administration, Writing – original draft, Writing – review & editing. **Judith P. Andersen:** Conceptualization, Methodology, Supervision, Writing – original draft, Writing – review & editing.

## Declaration of competing interest

The authors declare that they have no known competing financial interests or personal relationships that could have appeared to influence the work reported in this paper.

## Footnotes

1 Heart rate was measured on a separate device than HRV, which limits our ability to report concurrent HR and HRV metrics.
